# Welcome to the BSR Annual Meeting 2017

**DOI:** 10.5334/jbr-btr.1441

**Published:** 2017-11-18

**Authors:** Geert Villeirs

**Affiliations:** 1 UZ Gent, BE

**Keywords:** Belgian Society of Radiology, BSR, Annual Meeting

Dear colleagues,

It is again my great pleasure to welcome you to the BSR Annual Meeting.

The attendance rate at our meetings has been steadily increasing, with over 350 participants two years ago at the Diamant Conference & Business Center in Brussels and almost 500 participants last year at the Steigenberger Wiltcher’s Hotel.

As in previous editions, this year’s program will feature parallel sessions organized by the BSR Scientific Council and the Young Radiologist Section, both focusing on neuroradiology and paediatric radiology. National and international experts in the field will share their expertise and opinion on these topics in both cutting-edge lectures and refresher courses. After lunchtime, a short plenary session will provide some background about the current status and future directions in Belgian radiology, as 2017 has been quite a turbulent year both in budgetary and (MRI) programmatic terms. This year’s program will again be concluded with a “Clash of the Titans”, a rather new but meanwhile largely appreciated feature of our Annual Meeting.

I gladly take the opportunity to particularly thank Professor Raymond Oyen, President of the Scientific Council, and Drs. Rita Lopes de Rosário and Naïm Jerjir, Presidents of the Young Radiologist Section, and all others involved in the organization of this Annual Meeting, for a great job well done.

I wish you all a both interesting and pleasant BSR Annual Meeting 2017!

Warm regards,

Prof. Dr. Geert Villeirs

BSR President

